# Lifestyle-Induced Redox-Sensitive Alterations: Cross-Talk among the RAAS, Antioxidant/Inflammatory Status, and Hypertension

**DOI:** 10.1155/2021/3080863

**Published:** 2021-10-25

**Authors:** Renáta Szabó, Denise Börzsei, Alexandra Hoffmann, Zelma Nadin Lesi, Rudolf Gesztelyi, Béla Juhász, Gábor J. Szebeni, Jasmin Osman, Judith Sebestyén, Arnold Nagy, Sándor Szegedi, Csaba Varga, Anikó Pósa

**Affiliations:** ^1^Department of Physiology, Anatomy and Neuroscience, Faculty of Science and Informatics, University of Szeged, Szeged H-6726, Hungary; ^2^Interdisciplinary Excellence Centre, Department of Physiology, Anatomy and Neuroscience, University of Szeged, Szeged, Hungary; ^3^HR-Pharma Ltd., Szeged, Hungary; ^4^Department of Pharmacology and Pharmacotherapy, University of Debrecen, H-4032 Debrecen, Hungary; ^5^Laboratory of Functional Genomics, Institute of Genetics, Biological Research Centre, Hungarian Academy of Sciences, Szeged H-6726, Hungary; ^6^South-Pest Hospital Centre, National Institute for Infectology and Haematology, Department of Burns and Plastic Surgery, Budapest H-1097, Hungary; ^7^NAGYKUN-HÚS Ltd., Kunhegyes, H-5340, Hungary

## Abstract

The development and progression of hypertension are closely linked to an unhealthy lifestyle; however, its underlying mechanisms are not fully elucidated. Our aim was to assess the effects of diet and exercise on the elements of the renin–angiotensin–aldosterone system (RAAS), redox-sensitive parameters, and the expression of the vascular tone regulator endothelial nitric oxide synthase (eNOS). Male control Wistar-Kyoto (WKY) and stroke-prone spontaneously hypertensive (SHRSP) rats were randomized based on the type of diet (standard chow, high-fat diet: HT, and fructose-enriched diet: HF) and exercise (voluntary wheel-running exercise or lack of exercise). After 12 weeks of experimental period, the concentrations of the RAAS elements, myeloperoxidase (MPO) activity, tumor necrosis factor alpha (TNF-*α*) concentrations, levels of superoxide dismutase (SOD) and glutathione (GSH), and expressions of extracellular signal-regulated kinase1/2 (ERK1/2) and phosphorylated ERK1/2 as well as eNOS were measured in the cardiac tissue of WKY and SHRSP rats. We found that the RAAS elements were overactivated under hypertension and were further elevated by HT or HF diet, while HT and HF diet enhanced MPO and TNF-*α* parameters as well as the expression of pERK1/2; SOD, GSH, and eNOS levels were decreased. These changes occurred in WKKY rats and reached the statistically significant level in SHRSP animals. 12 weeks of exercise compensated the adverse effects of HT and HF via alleviating the concentrations of the RAAS elements and inflammatory markers as well as increasing of antioxidants. Our findings prove that SHRSP rats are more vulnerable to lifestyle changes. Both the type of diet and exercise, as a nonpharmacological therapeutic tool, can have a significant impact on the progression of hypertension.

## 1. Introduction

Growing evidence indicates that oxidative stress caused by overproduction of reactive oxygen species (ROS) plays a crucial role in the development of cardiovascular diseases (CVDs), such as hypertension, arrhythmias, and coronary ischemia [1, 2]. ROS accumulation mediates various signaling pathways, which promote redox imbalance as well as disruption of the vascular tone regulation [3]. A number of studies revealed a very close relationship between oxidative stress and overactivation of the renin–angiotensin–aldosterone system (RAAS) [4]. Abnormal activity of the RAAS can be associated with oxidative stress, elevated levels of inflammatory markers, and vascular cell degeneration, as well as with activation of various signaling pathways (e.g., PI3K/Akt or mitogen-activated protein kinase (MAPK) signaling cascades) that eventually lead to an unfavorable cardiac outcome [5, 6]. Angiotensin II (Ang II) serves as the main final mediator of the RAAS, which enhances oxidative stress, impairs endothelium-dependent vasorelaxation, and, consequently, makes the heart more vulnerable to ischemic events [7].

According to the mounting evidence, overconsumption of high-energy food and lower energy expenditure caused by sedentary lifestyle result in an alarming increase in the overproduction of ROS and inflammatory markers [8, 9]. Without an appropriate prevention or therapeutic strategy, these pathological changes are able to accelerate the incidence of CVDs or exacerbate the existing symptoms. Among strategies that have been considered successful against CVDs, regular physical exercise has beneficial effect in the prevention and treatment of cardiac damages [10]. Previous studies proved that physical exercise protect the cardiovascular system via alleviating oxidative damage and inflammatory processes, promoting endothelial nitric oxide (NO) production, and normalizing elevated blood pressure [3, 11].

Although the pathogenic effects of oxidative stress and their exercise-induced alterations in light of the CVDs are widely studied, complexity of the various molecular and biochemical mechanisms and intracellular pathways require further investigations. Based on the great interest towards the effects of oxidative damage and the potential therapeutic approaches against CVDs, we set a goal to examine the underlying biochemical mechanisms of lifestyle changes in a stroke-prone spontaneously hypertensive rat (SHRSP) model and age-matched WKY controls. Here, we studied the exposure of the heart to high-fat and fructose-enriched diets on inflammatory profile, antioxidant defense mechanisms, the RAAS elements, and their consequence on endothelial nitric oxide synthase enzyme- (eNOS-) mediated vascular dysfunction. We hypothesized that physical exercise possesses beneficial effects on these changes; thus, voluntary wheel-running exercise was selected as a nonpharmacological therapeutic tool.

Results from our study can help us to understand the underlying mechanisms by which lifestyle changes, including diet and exercise, may modify prehypertensive or hypertensive conditions.

## 2. Materials and Methods

### 2.1. Animals and Experimental Protocol

**Figure 1:**
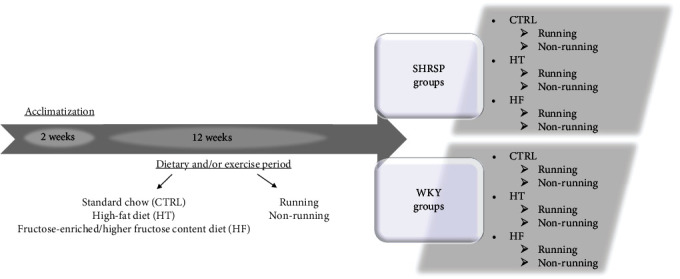
The experimental protocol of the study.

Five-week-old male WKY and SHRSP rats were used in this study. The rats were housed (Directive 2010/63/EU) in a temperature- (20–23°C) and light-controlled (12 : 12 hour light : dark cycle) room. After a 2-week acclimatization period, WKY and SHRSP rats were randomized based on the type of diet and physical exercise. The diets consist of standard chow (CTRL), high-fat diet (HT), or fructose-enriched/higher-fructose content diet (HF). HT diet was prepared by mixing the standard chow with 40% lard as the source of increased fat [12]. Rats in the HF groups were provided with 10% fructose-enriched standard chow [13]. At the same time with the dietary period, rats in the different dietary groups were further assigned either to sedentary or exercising groups. Voluntary wheel-running exercise was selected for that purpose to isolate the effects of exercise from the additional stress associated with forced exercise protocols [14]. Rats were placed into special cages fitted with a running-wheel and were allowed to free access to the wheel for 24 h/day. All animals were allowed free access to food and water. The health status of the animals was monitored during the experimental period. In our work, all efforts were made to minimize the number of animals as well as the animal suffering. At the end of the experimental dietary and/or running protocol, the rats were sacrificed, blood samples were taken, and the hearts were excised. Heart samples were clamped, milled, and stored at -80°C for further biochemical measurements. The experimental protocol and animal groups are presented in Figure 1.

The animal protocols were examined and approved by the Institutional Ethics Committee and were performed in accordance with the standards of the European Community guidelines on the care and use of laboratory animals (XX./983/2021).

### 2.2. Determination of Renin, ACE, Ang II, Aldosterone, and TNF-*α* Concentrations

A given amount of powdered heart tissue was homogenized with the corresponding amount of phosphate-buffered saline (PBS; pH 7.4). Samples were placed into the centrifuge tube (at 2500 rpm) for 20 minutes; then, supernatants were collected carefully. Standards were diluted according to the manual of the Enzyme-linked Immunosorbent Assay (ELISA) kits. Sample wells were tested with 10 *μ*l antibodies and 50 *μ*l streptavidin-HRP. Color development was evoked by chromogen solutions A and B and stop solution. At the end of the assay, absorbance (OD) was measured under 450 nm wavelength. Concentrations of renin, ACE, Ang II, aldosterone, and TNF-*α* were expressed as picograms per milligram protein, nanograms per milligram protein, picomoles per gram protein, nanograms per milligram protein, and picograms per milligram protein, respectively.

### 2.3. Determination of Total GSH + GSSG Content

Frozen heart tissues were homogenized in Homo buffer A (0.25 M sucrose, 20 mM Tris, and 1 mM dithiothreitol (DTT)) at first and then in Homo buffer B (0.1 M CaCl_2_, 0.25 M sucrose, 20 mM Tris, and 1 mM DTT). Samples were centrifuged at 21 450 × g for 30 minutes at 4°C, and the remaining clear cytosolic fraction was used for the enzyme assay. In a 96-well plate, the following reagents were mixed: 20 *μ*l 5,5′-dithiobis-2-nitrobenzoic acid (DTNB), 140 *μ*l nicotinamide adenine dinucleotide phosphate (NADPH), 10 *μ*l glutathione reductase, and 40 *μ*l sample. DTNB formation was determined at 405 nm, 10 minutes after the initiation of the reaction. GSH levels were expressed as nanomoles per milligram protein.

### 2.4. Measurement of Superoxide Dismutase Activity

Frozen hearts were homogenized in ice-cold 0.1 M Tris/HCL, pH 7.4 containing 0.5% Triton X-100, 5 mM *β*-ME, and centrifuged at 14 000 × g for 5 minutes at 4°C. The remaining supernatant was transferred to a clean tube and was kept on ice until the measurements. After this, the samples were pipetted as duplicates into the plate (each sample had its own blank modality). As described in the manual, WST solution, dilution buffer, and enzyme working solution were added to the wells; then, the plate was incubated at 37°C for 20 minutes. Output was measured at 450 nm with a microplate reader. Data were analyzed as follows:

(1) $$\begin{array}{c} \text{SOD activity }\left(\text{inhibition rate}\%\right)=\frac{\left(A_{\text{blank}1}-A_{\text{blank}3}\right)–\left(A_{\text{sample}}-A_{\text{blank}2}\right)\times 100}{\left(A_{\text{blank}1}–A_{\text{blank}3}\right)}. \end{array}$$

### 2.5. Determination of MPO Activity

Heart tissues were homogenized with PBS (pH 6.0) containing 0.5% hexadecyltrimethylammonium bromide. Homogenized samples were placed first in liquid nitrogen then in a water bath (37°C). These steps were repeated 3 times; then, the samples were centrifuged (15 000 × g for 15 min at 4°C) and supernatants were collected. The measurement was performed in a 96-well plate. 280 *μ*l of o-dianisidine diHCL along with 12 *μ*l of sample or standard (diluted from peroxidase) was pipetted into the plate. Cardiac MPO activity was measured under 490 nm wavelength after 59 seconds of shaking and expressed as microunits per milligram protein.

### 2.6. Western Blot Analyses for eNOS, p44/42 MAPK (ERK1/2), and Phospho-p44/42 MAPK (pERK1/2)

The tissue homogenates from the cardiac tissue were subjected to Western blot analyses for the determination of protein levels of eNOS, ERK, and pERK. The cardiac tissues were homogenized in ice-cold radio immunoprecipitation assay (RIPA) buffer. Homogenates were centrifuged at 12 000 rpm at 4°C for 10 min. Equal amounts of protein (50 *μ*g) were analyzed through electrophoresis (90 V/2 gels) on 8% polyacrylamide gel for eNOS and 10% gel for ERK1/2 and pERK1/2. Then, the proteins were transferred onto polyvinylidene fluoride membranes overnight at 25 V for eNOS and for 2.5 hours at 30 V for ERK1/2 and pERK1/2. Nonspecific binding was blocked by incubating 5% nonfat dry milk powder dissolved in TBS-Tween for 2 h a room temperature followed by 3.5 hours at 4°C for eNOS. Membranes for ERK1/2 and pERK1/2 were blocked in a solution of 5% bovine serum albumin (BSA) and TBS-Tween for 2 h at room temperature. Then, the blots were probed with 1 : 500 anti-eNOS mouse primary antibody overnight at 4°C in 1% milk powder and 1 : 1000 anti-ERK1/2 as well as anti-pERK1/2 rabbit primary antibodies overnight at 4°C in 5% BSA. The membranes were then incubated for 1 h at room temperature with 1 : 2500 either polyclonal rabbit anti-mouse horseradish peroxidase (Dako) for eNOS or polyclonal goat anti-rabbit horseradish peroxidase (Dako) for ERK1/2 and pERK1/2. For immunoblots, *β*-actin served as the loading control to show that similar amounts of protein were loaded in each lane (1 : 4000 anti-mouse primary antibody, 2 h, ab20272, Abcam, and 1 : 2000 polyclonal rabbit anti-mouse horseradish peroxidase, 1 h, Dako).

MagicMark XP Western Protein Standard (Invitrogen, Thermo Fisher Scientific, Waltham, MA, USA) was used for convenient protein determination. The standard consists of 9 recombinant proteins ranging in molecular weight from 20 to 220 kDa and allowed the identification of the proteins. The bands were visualized by Uvi Chemi Pro. and then analyzed by Quantity One software (Bio-Rad, Hercules, CA, USA).

### 2.7. Statistical Analysis

Normality of data and homogeneity of variances were verified with the Shapiro-Wilk test and Brown-Forsythe test, respectively. For comparison, three-way ANOVA with Tukey posttesting was used. Statistical significance for the difference of means was assigned into one of four categories: *p* > 0.05 (not significant), *p* < 0.05 (∗), *p* < 0.01 (∗∗), or *p* < 0.001 (∗∗∗). Statistical analysis was carried out with the use of GraphPad Prism 8.4.3 software.

## 3. Results

### 3.1. Determination of Cardiac Renin, ACE, and Ang II Concentrations

To determine whether lifestyle changes modulate the components of the RAAS, cardiac concentrations of renin, ACE, and Ang II were examined. It is clearly shown that the different factors, including rat strain, diet, and physical exercise, by itself, have a significant impact on the renin–ACE–Ang II axis; however, the interactions between different factors prove that the factors can influence each other.

**Figure 2:**
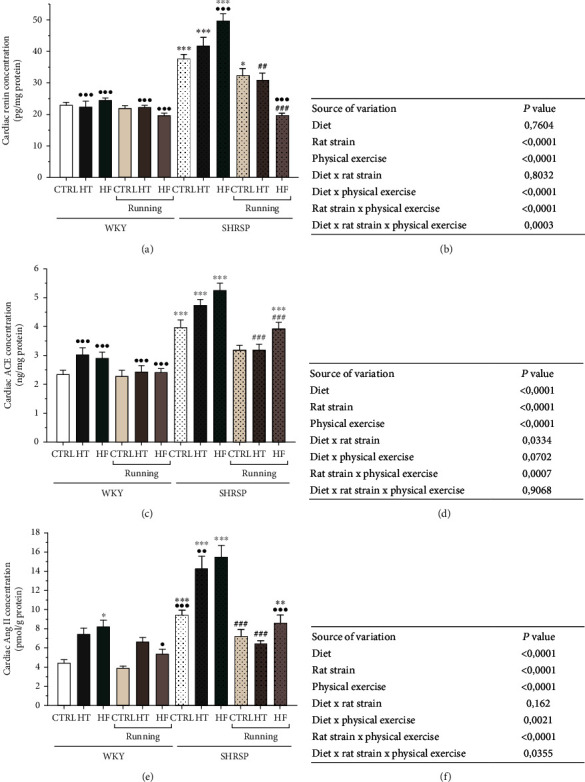
(a) The effects of lifestyle changes on cardiac renin concentration (expressed as pg/mg protein). Results are presented as means ± S.E.M., *n* = 5–6. (b) Statistical table of the individual effects and interactions of rat strain, diet, and physical exercise on cardiac renin concentration. (c) The effects of lifestyle changes on cardiac angiotensin-converting enzyme concentration (ACE, expressed as ng/mg protein). Results are presented as means ± S.E.M., *n* = 5–6. (d) Statistical table of the individual effects and interactions of rat strain, diet, and physical exercise on cardiac ACE concentration. (e) The effects of lifestyle changes on cardiac angiotensin II concentration (Ang II, expressed as pmol/mg protein). Results are presented as means ± S.E.M., *n* = 5–6. (f) Statistical table of the individual effects and interactions of rat strain, diet, and physical exercise on cardiac Ang II concentration. ^∗∗∗^*p* < 0.001, ^∗∗^*p* < 0.01, and ^∗^*p* < 0.05: statistical significance relative to the WKY/CTRL group; ^•••^*p* < 0.001, ^••^*p* < 0.01, and ^•^*p* < 0.05: statistical significance relative to the SHRSP/CTRL group; ^###^*p* < 0.001 and ^##^*p* < 0.01: statistical significance between the matching running and nonrunning groups.

As shown in Figure 2(a), lifestyle changes did not alter the cardiac renin concentration in WKY rats; however, SHRSP rats were more susceptible to diet- and exercise-induced changes. Cardiac renin concentrations were significantly elevated in all dietary groups of SHRSP rats compared with the WKY counterparts, and the greatest increase was observed in SHRSP/HF rats. Nevertheless, 12 weeks of physical exercise was able to compensate the unfavorable renin elevation in SHRSP rats. Our results show a triple interaction among the factors of rat strain, diet, and physical exercise (Figure 2(b)).

In accordance with the renin results, both ACE and Ang II changes verify that SHRSP rats are particularly sensitive to lifestyle changes. SHRSP rats exhibited significantly higher ACE concentrations compared with the matching WKY animals. Both HT and HF diet increased the ACE concentrations under hypertensive conditions, which was statistically significant in the SHRSP/HF group compared with the SHRSP/CTRL value (Figure 2(c)). Similar to the ACE changes, Figure 2(e) shows that Ang II, which is the main effector of the RAAS, was significantly elevated in SHRSP/HT and SHRSP/HF groups compared with the CTRL animals. 12-week-long voluntary wheel running effectively reduced the elevated ACE and Ang II values. Regarding SHRSP Ang II values, the elevated concentrations were reduced by almost half by exercise. While in the case of ACE values, we confirmed an interaction between rat strain and physical exercise, Ang II results showed a triple interaction among the factors of rat strain, diet, and physical exercise (Figures 2(d) and 2(f)).

### 3.2. Determination of Cardiac Aldosterone Concentration

To assess the impact of hypertensive condition, type of diet, and physical exercise on the complex RAAS activation, the other effector, aldosterone, was also examined. HT and HF diets resulted in elevation in both WKY and SHRSP rats, which was significant in the SHRSP/HF group compared with the SHRSP/CTRL group. We found that 12-week training period restored the adverse aldosterone levels, which approached the values of WKY animals.

**Figure 3:**
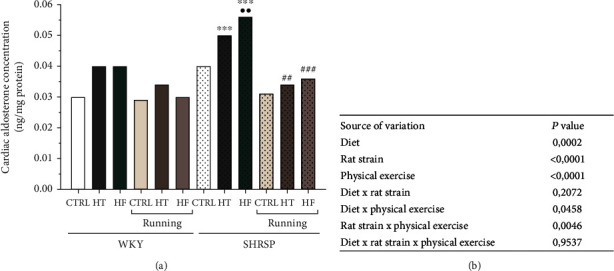
(a) The effects of lifestyle changes on cardiac aldosterone concentration (expressed as ng/mg protein). Result are presented as means ± S.E.M., *n* = 5–8. (b) Statistical table of the individual effects and interactions of rat strain, diet, and physical exercise on cardiac aldosterone concentration. ^∗∗∗^*p* < 0.001 and ^∗∗^*p* < 0.01: statistical significance relative to the WKY/CTRL group; ^•••^*p* < 0.001 and ^••^*p* < 0.01: statistical significance relative to the SHRSP/CTRL group; ^###^*p* < 0.001 and ^##^*p* < 0.01: statistical significance between the matching running and nonrunning groups.

Our results indicate that aldosterone changes are sensitive to hypertensive condition, type of diet, and physical exercise as well. Data are presented in Figures 3(a) and 3(b).

### 3.3. Measurement of Cardiac GSH + GSSG Content

**Figure 4:**
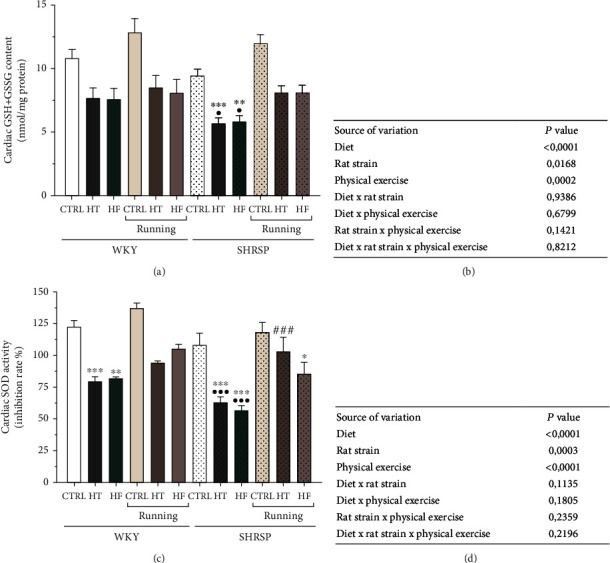
(a) The effects of lifestyle changes on cardiac GSH + GSSG content (expressed as nmol/mg protein). Result are presented as means ± S.E.M., *n* = 5–6. (b) Statistical table of the individual effects and interactions of rat strain, diet, and physical exercise on cardiac GSH + GSSG content. (c) The effects of lifestyle changes on cardiac superoxide dismutase activity (SOD, expressed as inhibition rate %). Result are presented as means ± S.E.M., *n* = 4–6. (d) Statistical table of the individual effects and interactions of rat strain, diet, and physical exercise on cardiac SOD activity. ^∗∗∗^*p* < 0.001, ^∗∗^*p* < 0.01, and ^∗^*p* < 0.05: statistical significance relative to the WKY/CTRL group; ^•••^*p* < 0.001 and ^•^*p* < 0.05: statistical significance relative to the SHRSP/CTRL group; ^###^*p* < 0.001: statistical significance between the matching running and nonrunning groups.

Compared with the CTRL rats, the consumption of HT or HF diet resulted in a decrease in cardiac GSH + GSSG content in WKY rats, which was statistically significant in SHRSP animals. 12 weeks of exercise did not cause a considerable elevation in the antioxidant capacity of WKY hearts; however, HT- and HF-induced reductions were compensated by exercise in SHRSP animals. Moreover, this improvement reached the values of the running WKY/HT and WKY/HF. As expected, the highest antioxidant GSH + GSSG content was measured in the hearts of running CTRL rats. Data are presented in Figures 4(a) and 4(b).

### 3.4. Measurement of Cardiac SOD Activity

Similar to the GSH + GSSG changes, cardiac SOD activity was significantly decreased as a result of HT and HF diet compared with their matching CTRL animals. In both Wistar and SHRSP animals, HT and HF diets produced a ~40% reduction of SOD activity compared with the CTRL values. The exercise-induced elevation in SOD activity, especially in the running SHRSP/HT and SHRSP HF groups, confirmed that exercise is able to improve the antioxidant defense mechanism in the heart of hypertensive rats. Our results show that all factors (rat strain, diet, and physical exercise) by itself had a significant impact on the SOD activity. Data are presented in Figures 4(c) and 4(d).

### 3.5. Measurement of Cardiac TNF-*α* Concentration

**Figure 5:**
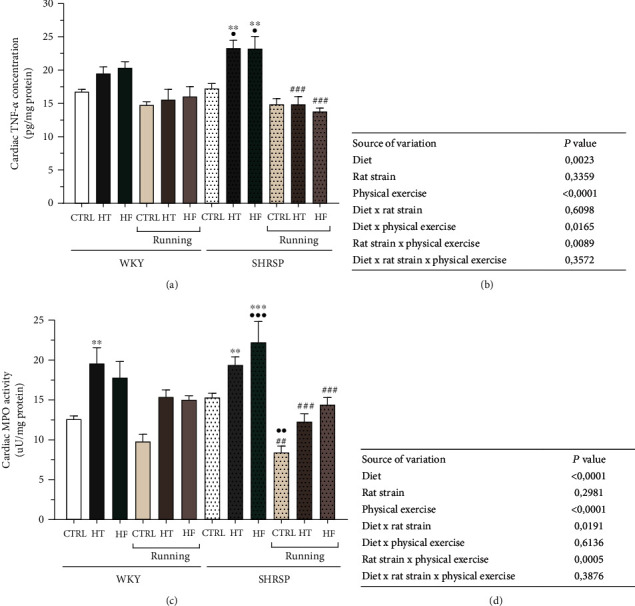
(a) The effects of lifestyle changes on cardiac tumor necrosis factor-alpha concentration (TNF-*α*, expressed as pg/mg protein). Result are presented as means ± S.E.M., *n* = 5–7. (b) Statistical table of the individual effects and interactions of rat strain, diet, and physical exercise on cardiac TNF-*α* concentration. (c) The effects of lifestyle changes on cardiac myeloperoxidase enzyme activity (MPO, expressed as *μ*U/mg protein). Result are presented as means ± S.E.M., *n* = 5–7. (d) Statistical table of the individual effects and interactions of rat strain, diet, and physical exercise on cardiac MPO activity. ^∗∗^*p* < 0.01 and ^∗^*p* < 0.05: statistical significance relative to the WKY/CTRL group; ^•••^*p* < 0.001, ^••^*p* < 0.01, and ^•^*p* < 0.05: statistical significance relative to the SHRSP/CTRL group; ^###^*p* < 0.001 and ^##^*p* < 0.01: statistical significance between the matching running and non-running groups.

To investigate the effects of lifestyle changes on inflammatory status, we determined the cardiac TNF-*α* concentration.  Figure 5(a) shows that the proinflammatory TNF-*α* values were enhanced in WKY rats fed with HT and HF diet and were further aggravated in SHRSP animals. Rats in SHRSP/HT and SHRSP/HF groups exhibited the highest TNF-*α* concentrations, which were mitigated by 12 weeks of exercise. Our statistical findings prove that diet and physical exercise significantly influenced the TNF-*α* values (Figure 5(b)).

### 3.6. Measurement of Cardiac MPO Activity

Our results provide further evidence that HT and HF diets have an unfavorable impact on inflammatory status of the heart, since the MPO values increased in both WKY and SHRSP rats fed with HT or HF diet. In SHRSP rats, voluntary wheel-running exercise resulted in a ~50% decrease in CTRL rats and a ~40% decrease in HT and HF groups. These findings were supported by the interaction between rat strain and physical exercise. Data are presented in Figures 5(c) and 5(d).

### 3.7. Evaluation of Cardiac ERK1/2 and pERK1/2 Expressions

**Figure 6:**
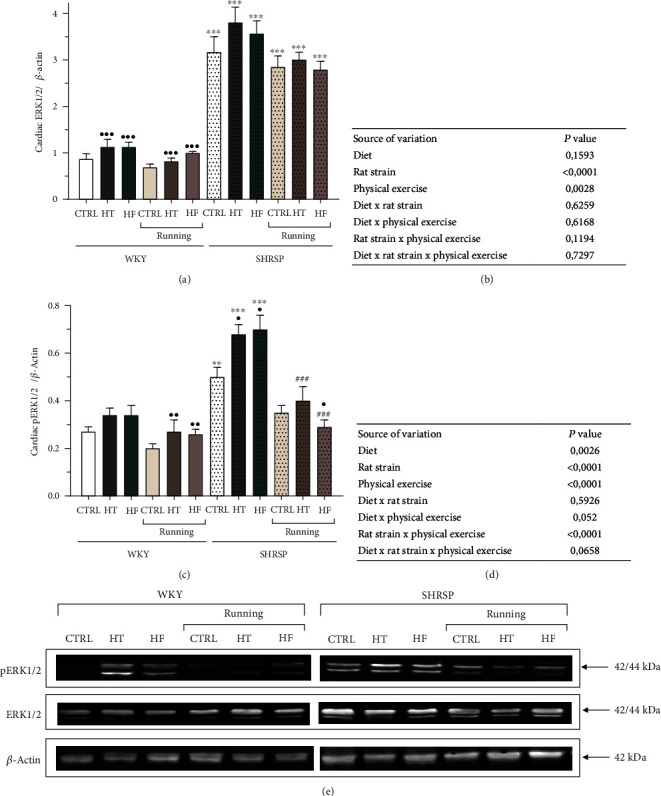
(a) The effects of lifestyle changes on cardiac extracellular signal-regulated kinase 1/2 expression normalized to *β*-actin (ERK1/2/*β*-Actin). Results are presented as means ± S.E.M., *n* = 5–6. (b) Statistical table of the individual effects and interactions of rat strain, diet, and physical exercise on cardiac ERK1/2/*β*-actin. (c) The effects of lifestyle changes on cardiac phosphorylated extracellular signal-regulated kinase 1/2 expression normalized to *β*-actin (pERK1/2/*β*-actin). Results are presented as means ± S.E.M., *n* = 5–6. (d) Statistical table of the individual effects and interactions of rat strain, diet, and physical exercise on cardiac pERK1/2/*β*-actin. (e) Representative Western blot images of the cardiac expressions of ERK1/2, pERK1/2, and *β*-actin. ^∗∗^*p* < 0.01 and ^∗^*p* < 0.05: statistical significance relative to the WKY/CTRL group; ^•••^*p* < 0.001, ^••^*p* < 0.01, and ^•^*p* < 0.05: statistical significance relative to the SHRSP/CTRL group; ^###^*p* < 0.001: statistical significance between the matching running and nonrunning groups.

Figures 6(a)–6(e) present that hypertensive condition, by itself, resulted in a significant increase in the expressions of ERK1/2 and pERK1/2 values in SHRSP animals compared with the matching WKY groups. As for the values of pERK1/2, we found that hypertensive condition resulted in a significant increase in pERK1/2 expression, which was further aggravated by HT or HF diet in SHRSP animals. Besides hypertensive condition and diet, physical exercise had a significant impact on pERK1/2. Our training protocol significantly improved the elevated pERK1/2 levels of SHRSP rats compared with the nonrunning counterparts.

### 3.8. Evaluation of Cardiac eNOS Expression

**Figure 7:**
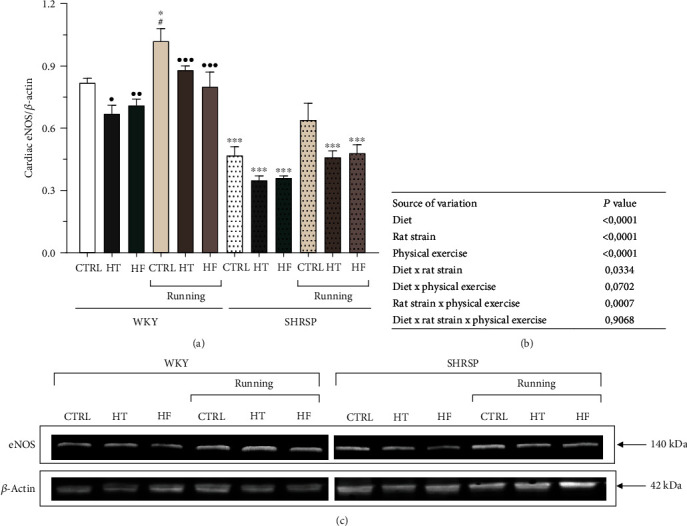
(a) The effects of lifestyle changes on cardiac endothelial nitric oxide synthase expression normalized to *β*-actin (eNOS/*β*-actin). Results are presented as means ± S.E.M., *n* = 4–5. (b) Statistical table of the individual effects and interactions of rat strain, diet, and physical exercise on cardiac eNOS/*β*-actin. (c) Representative Western blot images of the cardiac expressions of eNOS and *β*-actin. ^∗∗∗^*p* < 0.001 and ^∗^*p* < 0.05: statistical significance relative to the WKY/CTRL group; ^•••^*p* < 0.001, ^••^*p* < 0.01, and ^•^*p* < 0.05: statistical significance relative to the SHRSP/CTRL group; ^#^*p* < 0.05: statistical significance between the matching running and nonrunning groups.

To evaluate the potential effects of various diets and exercise on endothelial homeostasis, cardiac eNOS expression was evaluated. As shown in Figures 7(a)–7(c), SHRSP groups exhibited significantly lower eNOS expression compared with their matching WKY animals. The results clearly show that HT and HF diets resulted in a decrease in eNOS expression levels compared with their matching WKY groups. However, we found that 12 weeks of wheel-running exercise effectively compensated the reduced eNOS values in HT and HF groups and resulted in a further elevation in CTRL animals of both WKY and SHRSP strains.

## 4. Discussion

Hypertension is considered a major risk factor for global CVD morbidity and mortality; thus, its prevention and management are “top-priority” issues in the reduction of CVD risks and related healthcare burden. We set a goal to provide new insights into the impact of lifestyle (nutrition and physical exercise) on the underlying mechanisms of essential hypertension. In our study, control WKY and hypertensive SHRSP rats were used. SHRSP rat strain is a unique genetic model of severe hypertension, which was established from the strain of spontaneously hypertensive rats (SHR). Besides severe hypertension, SHRSP rats predispose to cardiovascular and metabolic diseases, nephropathy, and osteoporosis [15–17]; thus, they are suitable models for our examinations. In our study, we proved that unbalanced food consumption modified the RAAS elements (namely, renin, ACE, Ang II, and aldosterone), resulted in a redox imbalance, and eventually led to the impairment of vascular tone regulator eNOS. Secondly, our results also verify that physical exercise is an effective strategy to restore the redox balance and to favorable modify the RAAS-mediated pathways.

A large amount of evidence shows that oxidative stress is a hallmark of chronic diseases, such as hypertension [4, 7, 18]. Oxidative stress can be defined as a unifying mechanism, which occurs as a consequence of an altered balance between the production of ROS and antioxidant defense systems. The effects of ROS are mediated through redox-sensitive modulation of multiple regulatory pathways. Numerous studies provide ample evidence that perturbation of the RAAS plays a key role in the development and progression of CVDs [5, 19, 20]. Ang II has been well-recognized as the main effector of the RAAS for the regulation of blood pressure and cardiovascular homeostasis via type 1 Ang II receptor (AT_1_R), which can be found ubiquitously in the heart and blood vessels [7]. It has been previously described that Ang II can double ROS production, which clearly supports the fact that Ang II-mediated ROS is a trigger factor for further ROS generation and redox imbalance by other sources [21].

In accordance with the literature, we found that Ang II concentration was significantly increased in SHRSP/CTRL rats compared with the WKY/CTRL group and was further enhanced as a result of HT or HF diet. Additionally, SHRSP rats fed with HT or HF diet exhibited the highest Ang II values. Accumulating data suggest that HT diet increases the deposition of lipids not only into the adipose tissue but also into nonadipose tissues and induces deleterious effects in various tissues and organs. Pereira et al. found that HT, administered in SHR rats, increased lipid accumulation and resulted in a 50% reduction of the renal function [22]. In another study with SHR rats, Cao et al. found that HT diet with 58% fat content developed pathophysiological abnormalities, which were characterized by increased levels of blood cholesterol, inflammatory processes, and blood pressure along with an accelerated decline in cardiac function [23]. These results suggest that HT diet can be a trigger factor in the exacerbation of mechanisms underlying hypertension. Singh and Metha summarized that the pathways by which dyslipidemia leads to vascular disease may frequently overlap with Ang II. The RAAS can be upregulated by abnormal lipid levels or altered glucose/insulin levels as well, which leads to endothelial dysfunction and increased vascular superoxide production [24]. Our findings demonstrate that not only HT diet caused deleterious shifts, but also HF diet enhanced Ang II concentration. In accordance with our results, Gao et al. found that 10% of fructose-enriched diet resulted in overactivation of the RAAS elements [13]. Extensive clinical and experimental studies demonstrate that redox signaling is critically involved in cardiovascular pathophysiology and thus, the molecular basis of hypertension may be deeply rooted in shifts in the prooxidant/antioxidant balance [4, 25–27]. Redox homeostasis is tightly regulated by antioxidant enzymes, including SOD, GSH, and catalase as well as nonenzymatic antioxidants (e.g., bilirubin and uric acid). Under normal conditions, these protective mechanisms have evolved to prevent ROS accumulation. However, with dysregulation of Ang II signaling, the prooxidant/antioxidant balance leads to perturbation of antioxidant defense systems, which contributes to cardiovascular dysfunction, and predisposes to diseases, such as hypertension. In a previous work, Rosenbaugh *et al.* summarized the role of antioxidant therapies in CVDs and demonstrated that activation of SOD inhibits the pathological actions of Ang II-induced oxidative damages [28]. In transgenic mice deficient in antioxidant enzymes, such as SOD or glutathione synthase, vasoconstrictive mechanisms prevail. Our results show that both SOD and GSH antioxidant enzymes are significantly decreased due to administration of HT or HF diet compared with the CTRL groups, and these changes were correlated with the reduction of Ang II. While Ang II values in the hypertensive SHRSP rats, especially in the rats fed with HT or HF, were elevated, their antioxidant capacity was significantly dropped. We observed that cardiac SOD and GSH levels of SHRSP/HT and SHRSP/HF rats decreased by almost half compared with the SHRSP/CTRL group. It is well-established that physical exercise, as a nonpharmacological tool, plays an important role in attenuating pathological abnormalities in the cardiovascular system [29–31]. Most of these studies, which analyze the molecular and biochemical effects of exercise, often focus on one chosen mechanism or system to characterize cardiac alterations. Here, we examined the effects of exercise on the complex biochemical interactions of the RAAS system, redox-signaling mechanisms, and vascular tone regulation in both a normotensive WKY model and genetically modified, hypertensive SHRSP rats. Our results show that hyperactivity of the RAAS with imbalance between ACE-Ang II axes is a potent stimulus to disrupt the prooxidant/antioxidant balance; however, physical exercise was able to compensate these unfavorable changes. In line with the changes of Ang II concentrations, 12 weeks of wheel-running exercise significantly decreased the ACE concentration in all SHRSP running groups compared with the nonrunning SHRSP counterparts. Consequently, the levels of antioxidant SOD and GSH enzymes significantly improved in WKY and SHRSP running animals not only in the CTRL groups but also in rats fed with HT or HF diet, in which physical exercise was effective against the deleterious effects of the unbalanced diets. ACE is a membrane-bound enzyme which could be found in various cell types, such as endothelial cells. Beyond Ang II, ACE mediates effects on inflammation, oxidative stress, and blood pressure regulation [32, 33]. In a previous work, Silva et al. examined the independent effect of 8-week-long exercise on Wistar and SHR rats [34]. Similar to our findings, they observed exercise-induced deactivation of the cardiac Ang II, which was accompanied by an increase in the antioxidant capacity and a significant reduction in collagen content.

Besides alterations in the prooxidant/antioxidant balance, cardiac inflammatory parameters also can be significantly modified in response to lifestyle changes. Animal and human studies show that the RAAS plays a crucial role in the initiation and maintenance of inflammatory processes [35]. The RAAS activates ROS production and contributes to proinflammatory responses in the arteries, heart, and kidney by regulating the expression of cytokines and chemokines [5]. TNF-*α* is a proinflammatory cytokine, which is regulated by the NF-*κ*B redox signaling pathway. Increased proinflammatory cytokines activate the NF-*κ*B signaling pathway, and activation of NF-*κ*B, in turn, further upregulates cytokine production. It has been proven that exposure of the vasculature and myocardium to unfavorable carbohydrate or lipid content contributes to chronic low-grade inflammation. Our results clearly show that either HT diet with 40% lard content or HF diet with 10% fructose enrichment significantly elevated the cardiac TNF-*α* concentration in both WKY and SHRSP rats. However, 12 weeks of wheel-running exercise effectively attenuated the pathological TNF-*α* values. Similar to the changes in TNF-*α* concentrations, we observed a similar tendency in MPO enzyme activity. The leukocyte-derived MPO produces a cascade of ROS, including hypochlorous acid, which may ultimately lead to lipid peroxidation and NOS enzyme inhibition [36]. Under inflammatory conditions, MPO is linked to the RAAS, which aggravates endothelial dysfunction [37]. Previous work from our laboratory verified that both TNF-*α* and MPO enzyme are susceptible to lifestyle changes [12, 38]. Elevation in inflammatory parameters leads to the disruption of the vascular tone regulation, thus making the heart more vulnerable to the development of cardiovascular disorders, such as ischemic/reperfusion injury and hypertension [39, 40]. Increased ROS and inflammatory state shift the action of the endothelium towards reduced vasodilation and uncoupling of eNOS. L-arginine and tetrahydrobiopterin (BH_4_) are two essential cofactors during the action of eNOS; thus, their deficiencies are associated with uncoupling of L-arginine–NO pathway resulting in a decreased formation of NO [41]. The balance between NO and Ang II is an important regulator of the sympathetic tone; thus, NO reduction loses its role in antagonizing the effects of Ang II, and as a consequence, ROS production becomes overwhelming [41]. Our results clearly show that cardiac eNOS expression in SHRSP rats was only half the values of the WKY animals. While HT and HF diet contributed to the further reduction of eNOS expression, 12 weeks of voluntary exercise was able counteract the unfavorable changes.

Like Ang II, aldosterone is another effector molecule of the RAAS, whose receptors are present in the cardiac myocytes, endocardial endothelial cells, and cardiac fibroblasts. In a previous work, Takeda et al. summarized that aldosterone can be controlled by Ang II and that it participates in the pathological changes of the cardiovascular system in correlation with Ang II. Furthermore, they confirmed an increase in the aldosterone synthase activity in the hearts of SHRSP rats, which suggests that the local synthesis of aldosterone is increased in the cardiovascular system of the SHRSP rats [42]. High aldosterone level has been associated with myocardial ischemia, reduced coronary blood flow, and cardiac fibrosis through increased oxidant signaling and inflammation. The mechanisms by which aldosterone mediates these effects are due to the activation of ERK1/2 and NF-*κ*B [43, 44]. ERK is a member of the MAPKs family and is identifiable as two types: ERK1 and ERK2, which have an impact on cardiovascular homeostasis. We reported that both HT and HF diet are major triggers for ERK1/2 phosphorylation, since the pERK expression was significantly increased in SHRSP/HT and SHRSP/HF rats compared with their matching controls. However, physical exercise exerted beneficial influence on pERK 1/2 expression through exercise-mediated anti-inflammatory and antioxidant mechanisms. There is increasing literature suggesting the importance of the relationship between Ang II and aldosterone. These RAAS effectors either synergistically or via a cross-talk of their receptors may modulate signaling pathways, such as ERK1/2 [45].

## 5. Conclusion

In conclusion, recent findings prove that the SHRSP strain is particularly vulnerable to lifestyle changes. Both fat- and fructose-enriched diets serve as major triggers for RAAS-mediated cardiac damages in WKY and especially in SHRSP rats. HT and HF diets contributed to the disruption of prooxidant/antioxidant balance and inflammation. Exposure of the heart to decreased antioxidant defense systems as well as increased ROS and inflammatory parameters (TNF-*α* and MPO) overactivate the RAAS and ERK1/2 redox signaling mechanisms that progress to vascular dysfunction. Although the HT- and HF-mediated unfavorable effects have already occurred in WKY animals, changes in SHRSP rats suggest that both HT and HF exacerbate the RAAS-mediated mechanisms under hypertension. Voluntary wheel-running exercise targets antioxidant, inflammatory, and consequently RAAS-mediated mechanisms; thus, it seems likely that exercise is a potential nonpharmacological tool to manage hypertensive or hypertension-related conditions.

## 6. Limitation of the Study

Our study is not without limitations. Even though the underlying biochemical mechanisms of lifestyle-induced alterations are well-characterized in our study, the blood pressure of the rats was not analyzed. In order to discuss our biochemical findings with the interpretation of the underlying mechanism in light of the blood pressure changes, we used an inbred genetic model of hypertension.

## Data Availability

All data used to support the findings of this study are included within the article.

## Supplementary Materials

Supplementary Materials The effects of HT/HF diet and exercise on the RAAS-mediated inflammatory and antioxidant status as well as vascular homeostasis in WKY and SHRSP rats. eNOS: endothelial nitric oxide synthase; GSH+GSSG: reduced+oxidised glutathione; HF: fructose-enriched diet; HT: high-fat diet; MPO: myeloperoxidase; pERK: phosphorylated extracellular signal-regulated kinase1/2; RAAS: renin–angiotensin–aldosterone system; SHRSP: stroke-prone spontaneously hypertensive rat; SOD: superoxide dismutase; TNF-*α*: tumor necrosis factor-alpha; WKY: Wistar-Kyoto rat.
