# Supplementary figures and images for: Lifestyle-Induced Redox-Sensitive Alterations: Cross-Talk among the RAAS, Antioxidant/Inflammatory Status, and Hypertension
Source: Oxid Med Cell Longev. 2021 Oct 25;2021:3080863. doi: 10.1155/2021/3080863 (PMC8560269; doi:10.1155/2021/3080863)

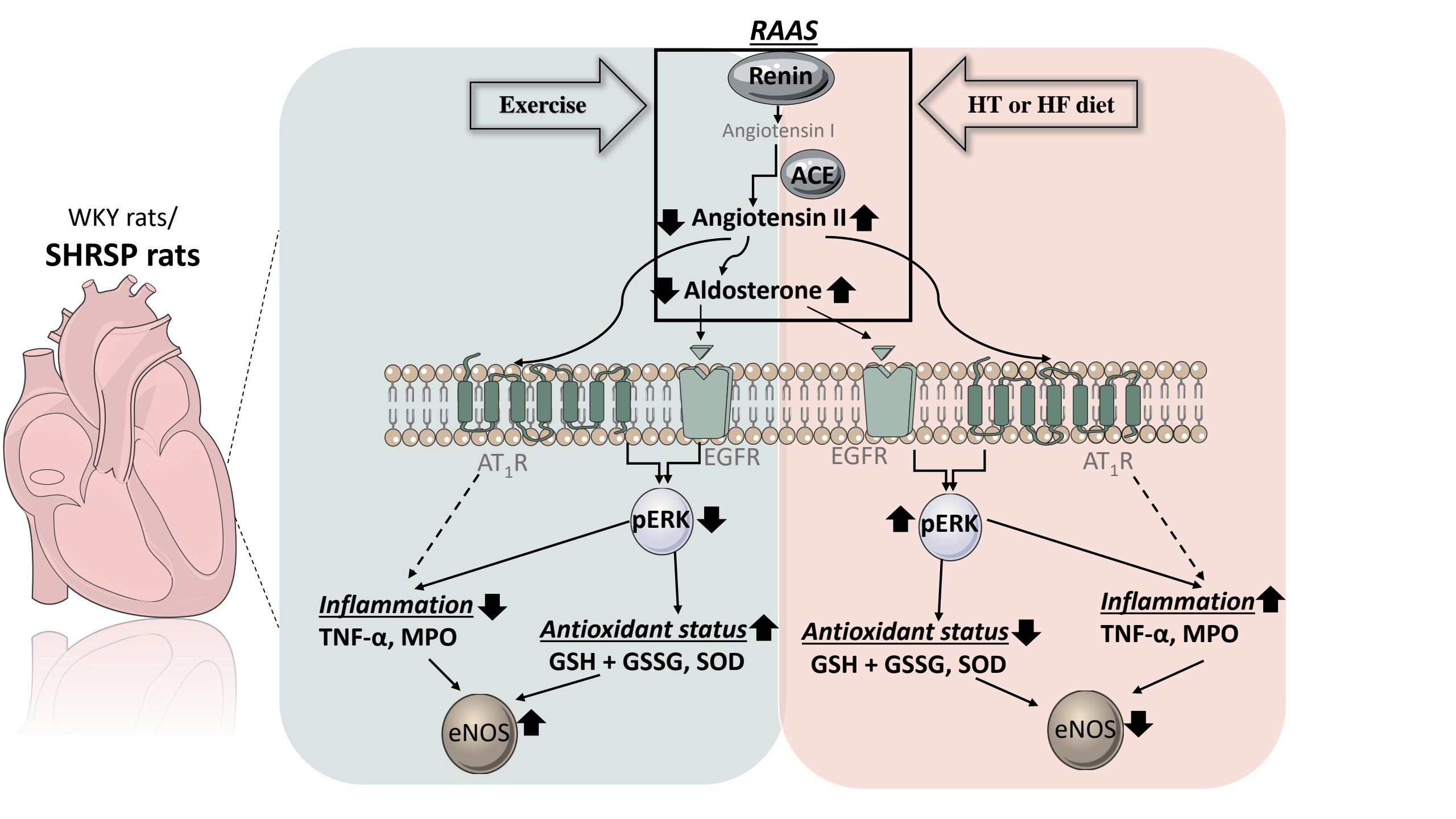

Supplement: Supplementary Materials — The effects of HT/HF diet and exercise on the RAAS-mediated inflammatory and antioxidant status as well as vascular homeostasis in WKY and SHRSP rats. eNOS: endothelial nitric oxide synthase; GSH+GSSG: reduced+oxidised glutathione; HF: fructose-enriched diet; HT: high-fat diet; MPO: myeloperoxidase; pERK: phosphorylated extracellular signal-regulated kinase1/2; RAAS: renin–angiotensin–aldosterone system; SHRSP: stroke-prone spontaneously hypertensive rat; SOD: superoxide dismutase; TNF-α: tumor necrosis factor-alpha; WKY: Wistar-Kyoto rat. [file 3080863.f1.pdf]
